# Exploring the concept of a “friendly relationship to death” in geriatric patients

**DOI:** 10.1186/s12877-025-06670-6

**Published:** 2025-12-19

**Authors:** Zoi Raboti, Alexander Rösler, Reinhard Lindner, Jürgen Gallinat, Brooke C. Schneider

**Affiliations:** 1 Department of Psychiatry and Psychotherapy, University Medical Center Hamburg-Eppendorf, Martinistraße 52, Hamburg, 20251 Germany; 2 Agaplesion Bethesda Hospital Bergedorf, Geriatric Clinic, Glindersweg 80, 21029 Hamburg, Germany; 3 Department of Social Work and Social Welfare, University of Kassel, Arnold-Bode- Str. 10, 34127 Kassel, Germany

**Keywords:** Death attitudes, Death acceptance, Fear of death, End of life, Dying, Life satisfaction, Suicidality, Older adults, Successful aging, Resilience

## Abstract

**Background:**

Positive attitudes toward death are common among geriatric patients, yet their distinction from mental illness and suicidality remains insufficiently understood. This study sought to characterize the concept of a “friendly relationship to death” (FRD), including its frequency in a geriatric sample and identifying variables from the geriatric assessment associated with a FRD.

**Methods:**

A cross-sectional study was conducted in a geriatric unit, surveying *N* = 99 patients aged 65 years and older. Attitudes toward death were assessed using The Multidimensional Orientation Toward Dying and Death Inventory* (*MODDI-F*)*, supplemented by one question addressing a FRD (“I have a friendly relationship to death.”), as well as eight additional questions on attitudes toward death, dying and religiosity/spirituality*.* Furthermore, depression, suicidality, disease burden, frailty, and loneliness were evaluated using validated questionnaires.

**Results:**

A total of 70% of patients endorsed a FRD. A FRD was associated with greater acceptance of one’s own dying and death, lower fear and rejection of death, greater religiosity/spirituality, and a stronger sense of “satisfied hunger for life.” A FRD was not associated with depression, suicidality, overall disease burden, frailty or loneliness.

**Conclusion:**

Among geriatric patients, a FRD appears to prevail, extending beyond a neutral acceptance of death’s inevitability and existing independently of suicidality, depression, frailty, or disease burden. Understanding a FRD may help contextualize older individuals’ acceptance of aging and could impact geriatric treatment outcomes.

**Supplementary Information:**

The online version contains supplementary material available at 10.1186/s12877-025-06670-6.

## Background

Cornerstones of geriatric care include discussions not only regarding interventions to treat and diagnose illness, extend the lifespan or maintain quality of life, but in line with national guidelines, should also include conversations about wishes and concerns regarding death and dying [1, 2]. However, the assessment of topics such as preferences surrounding death or suicidal thoughts in older adults often prove to be challenging. Particularly, distinguishing between passive death wishes or feeling of satiety regarding a life lived versus the intent to actively end one’s life (e.g., suicidality) is not always possible [3–5]. As such, it is not surprising that many geriatricians hesitate to actively broach these topics with their patients. When they do, clinicians then face the difficulty of interpreting and reacting to statements regarding death wishes and possible suicidal ideation [6]. Given that older adults with death wishes often seek contact with medical professionals but do not explicitly express their desire to die, improved knowledge regarding differentiation between active wishes to die versus a passive readiness to die is crucial and may improve patient-doctor communication [4].

Positive attitudes toward death, specifically among geriatric patients in clinical settings, have been less frequently examined [7] and it remains unclear how theoretical concepts of death acceptance may translate to the clinical context. Specifically, many geriatric patients express a passive readiness for or even a welcoming of death, which could be described as a “friendly relationship to death” (FRD). These expressions seem to be associated with a sense of a completed life and suggest an affective warmth toward death itself, as a familiar and non-threatening presence rather than an abstract inevitability. Although aspects of a friendly relationship with death have been previously mentioned [8], a FRD could differ from current concepts of death acceptance.

Research on death and dying has primarily focused on death anxiety, viewing death as an unavoidable but unwanted aspect of life and aging that can be minimized through defense mechanisms [9]. Although acceptance of one’s own dying and death has also been discussed as an important aspect of aging [7, 10], this concept has received less attention and is often absent from positive aging models [9]. Indeed, some form of acceptance of one’s own finitude has been shown to be the dominant attitude toward death in later life [11, 12]. For example, the Three-Component Model of Death Acceptance by Wong [13], assessed through the Death Attitude Profile-Revised (DAP-R) has been widely utilized in research on death attitudes. This model distinguishes three forms of death acceptance: (1) neutral acceptance, in which death is perceived as an “integral part of life, that is neither feared nor welcomed”, (2) an approach acceptance, in which death is embraced with the hope of a happy afterlife, often grounded in religious beliefs, and (3) an escape acceptance, in which death is viewed as a preferable alternative to a life characterized by pain and misery. A FRD would differ from these acceptance constructs given the apparently affectively positive (friendly) connotations older adults have with death (differing from a neutral acceptance) along with the absence of a sense of “escaping” an unbearable present life or longing for a glorious afterlife (differing from escape and approach acceptance)*.*

Wittkowski [14] introduced the Multidimensional Orientation toward Dying and Death Inventory (MODDI-F), which considers attitudes toward the process of dying, including potential pain and physical challenges in addition to the prospect of death itself as the end of personal existence. Importantly, Wittkowski’s unidimensional acceptance of dying and death, which is most similar to Wong et al.‘s concept of neutral acceptance, implies an acknowledgment of the natural and inevitable nature of death without an affective component [15].

Interestingly, Wong [16] later posited that neutral acceptance is not a unitary construct but rather consists of two components: the rational recognition of the naturalness of death on the one hand along with an affective, meaning-making component, such as „identifying oneself with culture”, “completing one’s mission in life”, and “leaving a legacy” on the other hand. Extending upon this, Wong’s Meaning Management Theory (MMT) [16] suggests that death acceptance is strengthened when death is perceived as a meaningful part of life and serves as an impulse for self-actualization (rather than a constant threat). At the same time, anthropological perspectives have highlighted the increasing desacralization of death [17] and its relegation to medical institutions. Thus, older adults, faced with a heightened awareness of life’s finitude may cultivate a readiness for death that integrates both a rational recognition and existential meaning. This is in line with findings that death acceptance tends to reach its highest levels in later life [18–20]. To the best of our knowledge, these alternative views of death acceptance—marked by a self-actualized readiness for death without the need to hasten it—have not been incorporated into existing scales but may be reflected in the concept of a FRD*.*

With increasing age, the likelihood of being confronted with mortality (mortality salience), such as through disabling medical conditions or the loss of loved ones, naturally increases [21]. Whereas older adults with multimorbidity, loneliness or cognitive impairment remain at a higher risk for depression and suicidality [22, 23], subjective well-being in older age generally remains relatively stable or even increases into old age [24]. One explanation for this lies in resilience, understood as adaptability, effective coping, and psychological strength, which helps sustain life satisfaction and supports successful aging despite adversity [25]. Acute reminders of one’s own finitude, such as life-threatening illnesses can initially cause fear, but can also trigger adaptive processes that foster resilience and ultimately greater death acceptance [26–29]. For example, among cancer patients, acceptance of death increased with illness progression [30]. Further protective factors against depression and suicidality in old age include social support and spirituality [18, 31] *– *defined broadly as orientations that transcend the individual’s immediate reality, whether through connection to nature, humanity or a divine presence [32]. These factors may be especially relevant for geriatric patients coping with illness, and could play a role in shaping a FRD*.*

Taken together, the primary aim of the current study was to characterize the concept of a FRD among older adults on a geriatric unit including measuring prevalence and identifying variables typically included in geriatric assessments, such as depressive symptoms, suicidality, frailty, functional status, and illness burden, which may be associated with death attitudes. We sought to examine whether the category of a “friendly attitude toward death” could be distinguished from existing concepts such as death acceptance as well as suicidality and to examine associations between a FRD and a validated scale of death attitudes.

## Methods

### Study design

Patients hospitalized in a clinic for geriatrics between March 2022 and June 2023 were included in the cross-sectional data collection. The clinic for geriatrics has 60 beds, with 62.9% of the patients being female. Patients have a mean age of 83.2 years (SD 6.8). At admission, 61.2% live alone, the mean Barthel Scale [33] is 44.5 (SD 14.4) and the mean Mini Mental Status Examination (MMSE) [34] score is 22.8 (SD 6.0). The average length of stay is 18.5 days (SD 6.7) with an in-hospital mortality of 5.7%. Though most patients are severely ill, palliative cases are usually not admitted. Main admission diagnoses include internal medicine (e.g., COPD, sepsis, heart failure), neurological (e.g., stroke, hemorrhage, delirium, Parkinson’s disease), trauma surgery (e.g., fractures of femur, pelvis, spine), and other surgical procedures, mainly tumor operations.

Patients were included in the study if they (1) were at least 65 years old, (2) had a MMSE score of at least 23 points as reported in the medical record at the time of admission to the geriatric clinic, (3) did not have a legal guardian, (4) were able to speak German adequately for study participation, (5) provided informed consent, and (6) expressed readiness to complete the study questionnaires.

### Ethical aspects

This study was conducted under the 1964 Declaration of Helsinki, its subsequent amendments. Participants were informed about the purpose and scope of the study as well as the benefits and conditions of confidentiality. Furthermore, they were informed that they could withdraw their consent to participate in the study at any time without any adverse consequences. All participants provided signed informed consent. The Ethics Committee of the Hamburg Medical Association (Ärztekammer) approved the study (Ethics vote 2021–200275-BO-ff).

### Data collection

Sociodemographic variables and some clinical data (i.e., MMSE, Depression in Old Age Scale [DIA-S], Barthel Scale) [35] were collected during clinical interviews by trained geriatricians as part of the standard geriatric assessment, which all patients complete upon admission to the clinic (see descriptions below). Participants additionally completed the Multidimensional Orientation toward Dying and Death Inventory (MODDI-F) [36], including self-administered supplementary questions, the Beck Scale for Suicide Ideation (BSS) [37], and the 3-item version of the UCLA Loneliness Scale (T-ILS) [38]. Patients were assessed according to study team availability and once they were considered medically fit to participate, usually between day 3 and day 10 of their stay. After receiving general information about the study from a trained medical student, participants were surveyed at different time points during their stay in the geriatric clinic. They were either given the MODDI-F, the BSS and the T-ILS to complete alone or, if requested, completed the questionnaires with a medical student. In this case, the questions were read aloud, and special attention was paid to ensure privacy. The medical student subsequently assessed the Cumulative Illness Rating Scale for Geriatrics (CIRS-G) [39] and Clinical Frailty Scale (CFS) [40] based on information obtained from the clinical records.

## Measures

### Attitude toward dying and death

Attitudes towards dying and death were assessed using the German version of the Multidimensional Orientation toward Dying and Death Inventory (MODDI-F). It is a factor-analytically constructed scale that contains 47 items measuring acceptance, fear and rejection of dying and death. Importantly, on the MODDI-F, a distinction is made between the process of dying and the state of being dead, as well as between attitude towards one’s own dying and death and the dying and death of another person, resulting in eight factors. In this study, we only analyzed the dimensions referring to one’s own dying and death: “acceptance of one’s own dying and death” (AODD; 8 items, *„Somehow*,* the knowledge of my death is a part of my life that I view positively*.“), “fear of one’s own dying” (FODy; 8 items, *„I am afraid of dying a painful death one day.”*), “fear of one’s own death” (FODe; 6 items, *“The idea that I will never be able to think and experience anything after my death disturbs me.”*), and “rejection of one’s own death” (RODe; 5 items, *“Inwardly*,* I protest against the fact that I will be dead someday.”*) [41]. Items are answered on a 4-point Likert scale, with high values indicating higher acceptance or fear or rejection of dying and death. Cronbach’s alphas for the individual subscales used in this study were acceptable to good (alpha = 0.66 and 0.87). The subscale “*acceptance of one’s own dying and death*” showed a small but significant negative correlation with the subscale *“rejection of one’s own death”*, *r* [98] = − 0.227, *p* =.023, and with the subscale *“fear of one’s own death”*, *r* [98] = − 0.269, *p* =.007. The correlation with the subscale “*fear of one’s own dying*” was not significant, *r* [98] = 0.089, *p* =.381. Furthermore, the subscale “*fear of one’s own death*” showed a strong correlation with the subscale “*rejection of one’s own death*”, *r* [98] = 0.819, *p* <.001, and a significant but weaker correlation with the subscale “*fear of one’s own dying”*, *r* [98] = 0.357, *p* <.001. Additionally, *“fear of one’s own dying”* and *“rejection of one’s own death”* were also significantly correlated, *r* [98] = 0.364, *p* <.001.

### Friendly relationship to death (FRD)

Additional items regarding a FRD, death, dying and spirituality (see below also) were initially developed by two of the authors (a geriatrician and a specialist in geriatric psychosomatics and psychodynamic psychotherapy for older adults). Items were then presented to the other team members for review (psychiatrist with a specialty in aging, cognitive behavioral psychotherapist for older adults and a medical student). Phrasing of questions was adjusted slightly. No other piloting or validity/reliability checks were made prior to use of the items in the current study.

A FRD was assessed with one item: “I have a friendly relationship to death,” answered on a 4-point Likert scale, following the same principle as the MODDI-F. For group classification in Tables 1, 2 and 3, the response 0 = “completely disagree” was coded as “No” and the responses 1 = “agree somewhat”, 2 = “agree for the most part” as well as 3 = “agree almost totally” were coded as “Yes”.

**Table 1:** Sociodemographic characteristics of the total sample (*N* = 99) and in relation to participants’ “Friendly Relationship to Death”

		Friendly Relationship to Death		
	Total Sample *N* = *99* *M(SD)/N (%)*	Yes *(n* = 69*)* *M(SD)/N (%)*	No *(n* = 30*)* *M(SD)/N (%)*	Statistics
Age (years)	81.90 (6.54)	81.61 (6.61)	82.57 (6.42)	*U* = 930 *p* =.424
Gender				*χ*^*2*^ *[98] =* 1.38 *p* =.241
Male	44 (44.0%)	28 (40.6%)	16 (53.3%)	
Female	55 (56.0%)	41 (59.4%)	14 (46.7%)	
Marital Status				*χ*^*2*^ *[98] =* 0.002 *p* =.999
Married	43 (43.4%)	30 (43.5%)	13 (43.3%)	
Widowed	43 (43.4%)	30 (43.5%)	13 (43.3%)	
Single	13 (13.1%)	9 (13.0%)	4 (13.3%)	
Religious affiliation				*χ*^*2*^ *[97] =* 2.37 *p* =.499
Protestant	57 (57.6%)	42 (60.9%)	15 (50.0%)	
Catholic	5 (5.1%)	4 (5.8%)	1 (3.3%)	
None	37 (36.4%)	22 (31.9%)	14 (46.7%)	
Formal education ^a^				*χ*^*2*^ *[96] =* 4.18 *p* =.124
Lower secondary school	37 (37.4%)	22 (32.4%)	15 (50.0%)	
Higher secondary school	37 (37.4%)	30 (44.1%)	7 (23.3%)	
High school diploma	24 (24.2%)	16 (23.5%)	8 (26.7%)	
Living situation ^a^				*χ*^*2*^ *[95] = 2.24 p =.329*
Lives alone	42 (42.9%)	30 (44.1%)	12 (40.0%)	
Lives with relatives	52 (53.1%)	34 (50.0%)	18 (60.0%)	
Lives in care facility	4 (4.1%)	4 (5.9%)	0 (0.0%)	
Number of children				*χ*^*2*^ *[98] = 1.79 p =.616*
0	12 (12.1%)	10 (14.5%)	2 (6.7%)	
1	26 (26.3%)	19 (27.5%)	7 (23.3%)	
2	42 (42.4%)	27 (39.1%)	15 (50.0%)	
3 and more	19 (19.2%)	13 (18.8%)	6 (20.0%)	
Bodily care ^c^				*χ*^*2*^ *[98] = 1.23 p =.542*
Independent	56 (58.3%)	40 (60.6%)	16 (53.3%)	
Needs help	15 (15.6%)	11(16.7%)	4 (13.3%)	
Dependent	25 (26.0%)	15 (22.7%)	10 (33.3%)	

^*a* ^*N= 98*

^*b* ^*N= 96*

**Table 2:** Clinical characteristics of the total sample and in relation to participants’ “Friendly Relationship to Death”

Friendly Relationship to Death				
	Total Sample *N* = *99* *M(SD)*	Yes *(n* = *69)* *M(SD)*	No *(n* = 30*)* *M(SD)*	Statistics
MMSE	27.72 (1.58)	27.86 (1.54)	27.40 (1.65)	*U* = 872 *p* =.208
Barthel-Scale^a^	52.29 (12.70)	51.84 (12.81)	53.39 (12.62)	*U* = 941 *p* =.928
CFS				
Prior to admission	4.52 (1.49)	4.42 (1.52)	4.73 (1.41)	*U* = 906 *p* =.311
Current	5.38 (1.08)	5.36 (1.12)	5.43 (0.97)	*U* = 998 *p* =.754
CIRS	16.07 (4.53)	15.65 (4.67)	17.03 (4.09)	*U* = 850 *p* =.159
DIA-S	2.14 (2.40)	2.02 (2.30)	2.43 (2.64)	*U* = 944 *p* =.481
BSS	0.63 (1.43)	0.62 (1.50)	0.63 (1.27)	*U* = 1001 *p* =.732
T-ILS	3.19 (2.63)	3.42 (2.65)	2.67 (2.55)	*U* = 858 *p* =.175
Death attitudes (MODDI-F)				
Acceptance of one’s own dying and death (AODD)	21.46 (3.52)	22.22 (2.65)	19.73 (4.58)	***U *= 625 *p *<.001**
Rejection of one’s own death (RODe)	2.86 (2.53)	2.29 (3.27)	4.17 (3.80)	***U = 699 p*=.008**
Fear of one’s own dying (FODy)	15.81 (4.92)	15.78 (4.88)	15.90 (5.09)	*U* = 1035 *p* =.998
Fear of one’s own death (FODe)	3.45 (4.67)	2.83 (4.12)	4.90 (5.56)	*U* = 849 *p* =.143

^a^ *N* = *96*

MMSE = Mini Mental Status Examination

CFS = Clinical Frailty Scale

CIRS = Cumulative Illness Rating Scale

DIA-S = Depression in Old Age Scale

BSS = Beck Scale for Suicide Ideation

T-ILS = UCLA Loneliness Scale (3 item version)

MODDI-F = Multidimensional Orientation Toward Dying and Death Inventory

**Table 3:** Additional questions for the total sample and in relation to participants’ “Friendly Relationship to Death”

		Friendly Relationship to Death		
	Total Sample *N* = *99* *M(SD)*	Yes *(n* = 69*)* *M(SD)*	No *(n* = 30*)* *M(SD)*	Statistics
Additional questions				
1. “I often think about my death.”	0.93 (1.06)	1.00 (1.04)	0.77 (1.10)	*U* = 870 *p* =.180
2. “I think I will live longer than one year.”^a^	2.57 (0.88)	2.48 (0.96)	2.79 (0.63)	*U* = 798 *p* =.133
3. “I will be remembered fondly after my death.”^b^	2.55 (0.90)	2.57 (0.87)	2.50 (0.97)	*U* = 976 *p* =.762
4. “I would describe myself as a religious or spiritual person.”	1.42 (1.34)	1.61 (1.33)	1.00 (1.29)	***U *=771** ***p*=.033**
5. “My hunger for life is satisfied.”	1.63 (1.31)	1.80 (1.22)	1.23 (1.43)	***U *= 792** ***p*=.050**
6. “I do not expect any more from life.”^c^	0.87 (1.12)	0.88 (1.12)	0.83 (1.14)	*U* = 969 *p* =.796
7. “The thought of killing myself is not foreign to me.”^c^	0.74 (1.23)	0.66 (1.22)	0.93 (1.26)	*U* = 863 *p* =.141
8. “Here I wait for death without wanting or fearing it.”^c^	1.34 (1.29)	1.48 (1.31)	1.00 (1.20)	*U* = 802 *p* =.105

*Notes: 0 = do not agree at all*,* 1 = agree somewhat*,* 2 = agree for the most part*,* 3 = agree almost totally*

^*a*^ *N* *= 95*

^*b*^ *N* *= 97*

^*c* ^*N* *= 98*

### Additional questions on death and dying and spirituality

The remaining eight questions addressed death and dying as well as religiosity/spirituality: *“I often think about my death.”; “I think I will live longer than one year.”; “I will be remembered fondly after my death.”; “I would describe myself as a religious or spiritual *person*.”; “My hunger for life is *satisfied*.”; “I do not expect any more from life.”; “The thought of killing myself is not foreign to me.”; “Here I wait for death without wanting or fearing it.”*. These were answered following the same principle as the MODDI-F, allowing responses of 0 = “completely disagree” to 3 = “completely agree”.

### Depression

Depression was measured using the Depression in Old Age Scale (DIA-S). The scale contains 10 items, which can be answered with ‘no’ or ‘yes’, resulting in a possible range of 0 to 10 points. A score of 3 is suggestive of depression, whereas a score of 4 or more indicates that a clinically relevant depression is likely. This scale demonstrated good internal consistency (Cronbach’s α = 0.80).

### Suicidality

Suicidal thoughts were assessed using the first five questions of the German version of the Beck Scale for Suicide Ideation (BSS), which assess participants’ current wish to live or to die and screen for the presence of both passive and active suicide ideation during the past week. Each item can be scored from 0 to 2, with higher scores indicating a higher risk of suicide. Internal consistency for this scale was good (Cronbach’s α = 0.80).

### Loneliness

Loneliness was measured with the 3-item version of the UCLA Loneliness Scale (T-ILS). Validity with respect to the elderly population was tested in a comparison between nationally representative studies in Germany and the United States [42]. Each item can be scored from 1 to 3, resulting in a possible total score of 3 to 9 points, with 9 points indicating significant loneliness. Internal consistency for the T-ILS was good (Cronbach’s α = 0.83).

### Comorbidity

The Cumulative Illness Rating Scale for Geriatrics (CIRS-G) based on information drawn from the medical record was used to classify the patients’ burden of disease, paying particular attention to current impairments in everyday life caused by the illness. The CIRS-G considers a total of 14 organ systems and allows the severity of illness to be classified from 0 (= no damage) to 4 (= very severe damage) for each system, resulting in a minimum score of 0 and a maximum score of 56. The organ systems included are: (1) heart, (2) blood pressure and blood vessels, (3) hematopoietic and lymphatic system, (4) lungs and respiratory tract, (5) eyes and ear-nose-throat, (6) upper gastrointestinal tract, (7) lower gastrointestinal tract, (8) liver, gallbladder and pancreas, (9) kidneys, (10) urogenital tract, (11) musculoskeletal system and skin, (12) nervous system, (13) endocrine, metabolic disorders and mammary gland, and (14) psychological disorders.

### Frailty

Frailty was assessed using the Clinical Frailty Scale (CFS), which measures the frailty of older adults according to fitness level, ability to go for a walk, to shop independently and to bathe themselves, among other criteria, on a scale of 1 (very fit) to 9 (terminally ill) according to the geriatric assessment upon admission, patients’ self-reports, and the examiner’s evaluation. CFS was assessed based on functioning upon admission as well as functioning prior to hospitalization.

### Functioning

The Barthel Scale was used as a further systematic assessment method to record the independence of the patients in activities of daily living. Each of the ten categories is scored with a maximum of 10 points, such that a total score of 100 indicates maximum independence. In the context of the detailed geriatric assessment, additional information was collected (e.g., living situation, the need for assistance required for bodily care or the number of assistive devices used prior to hospitalization).

### Statistical analysis

SPSS (Version 29.0.2.0; IBM Corp., 2023) was used for statistical analysis. A general assessment of normality of the variables was conducted. Because most variables were not normally distributed, non-parametric tests were used for our analyses. First, we performed exploratory analyses using Spearman’s correlation to examine the relationships between the presence of a FRD and other variables, including disease burden, frailty, depression, and suicidality. In a second step, we aimed to evaluate whether group differences based on the presence and absence of a FRD could be detected. For this, we conducted Mann-Whitney *U* tests for continuous variables and cross-tabulations for categorical variables. Given that the aim of the study was to generate hypotheses regarding the construct of a FRD versus confirming previously established associations, significance testing was not adjusted for multiple comparisons*.*

### Sample size calculation

The main aim of this study was to examine the prevalence and correlates of a FRD. Given that a FRD has not been examined previously and data is lacking on death attitudes in samples of geriatric patients, sample size was calculated to detect correlations *r* > = 0.30 (Beta = 0.80; two-tailed), resulting in a sample size of 84 patients. To allow for possible between-group analyses, we decided to stop recruitment at 100 patients.

## Results

### Description of sample

One-hundred fifty-two (*N *= 152) patients were approached for the study, 113 agreed to participate, and 100 provided valid responses. One patient did not provide a response to the item pertaining to a FRD so that *N*= 99 cognitively intact, older adults ages 65 to 99 years (*M *= 81.9, *SD* = 6.5) were included in the study. Sociodemographic characteristics of the sample are presented in Table 1. Slightly more than half were women (56.0%) and the majority lived at home, either alone (42.9%) or with relatives (53.1%) prior to admission. Patients were admitted for conditions of internal medicine (heart failure, pneumonia, COPD, gastrointestinal bleeding, sepsis; *N *= 39), trauma/orthopedic issues (fractures of the spine, pelvis, femur, or forearm; *N* = 31), neurological disorders (cerebral infarctions or hemorrhages, Parkinson’s disease; *N* = 19), and general surgical problems (tumor surgeries, hepatobiliary infections; *N* = 11). Four patients who participated in the study died during their hospital stay. Further sociodemographic aspects can be found in Table 1.

Patients demonstrated on average “mild frailty” according to the CFS with a moderate to high level of illness burden on the CIRS-G and minimal depressive symptoms according to the DIA-S. On the MODDI-F, whereas both death acceptance (*M* = 21.46*)* and fear of one’s own dying (*M = *15.81*)* were rather high, participants were less likely to endorse fear and/or rejection of their own death. Detailed results of all clinical assessments can be found in Table 2.

### Friendly relationship to death

Responses to additional questions are provided in Table 3. A total of 70% (*n* = 69) of respondents agreed with the statement “*I have a friendly relationship to death*” to varying extents: 25% agreed with this statement “somewhat”, 15% indicated that they agreed with that statement “for the most part” and 29% indicated that they “almost totally” agreed. In contrast, 30% rejected this statement completely indicating that they “do not agree at all”. Tables 1, 2 and 3 compare these two groups in terms of sociodemographic and clinical characteristics.

Unadjusted correlation analyses are also reported in the Supplemental Information. A significant moderate association was found between a FRD and the MODDI-F subscale “acceptance of one’s own dying and death” (*r*_s_ [97] = 0.43; *p* <.001), such that individuals who endorsed greater death acceptance were also more likely to endorse having a FRD. In contrast, there was a significant negative correlation between a FRD and the MODDI-F subscales “fear of one’s own death” (*r*_s_ [97] = − 0.22; *p* =.030) and “rejection of one’s own death” (*r*_s_ [97] = − 0.33; *p* <.001). Two weaker significant associations were found between a FRD and the statements “*I would describe myself as a religious or spiritual person.*” (*r*_s_ [97] = 0.24; *p* =.015) and “*My hunger for life is satisfied.*” (*r*_s_ [97] = 0.20; *p* =.048). Depression (DIA-S) tended to correlate negatively with a FRD (*r*_s_ [97] = − 0.19; *p* =.055), however, did not reach significance. No further significant correlations between a FRD and all other variables of interest were found (e.g., suicidal ideation (BSS), comorbidity (CIRS), frailty (CFS), activities of daily living (Barthel Score), mental status (MMSE), loneliness (T-ILS), fear of dying (MODDI-F) and all other self-generated additional questions).

Regarding group differences between participants with vs. those without a FRD for unadjusted analyses, significant differences were detected for the MODDI-F subscale “acceptance of one’s own dying and death” as well as “rejection of one’s own death” such that individuals with a FRD endorsed greater acceptance and less rejection. Individuals with a FRD were also significantly more likely to endorse being spiritual and that their hunger for life is satisfied.

## Discussion

This study sought to identify attitudes toward death among geriatric patients, particularly with regard to the concept of a FRD. This work is borne out of interactions with geriatric patients who report a “readiness to die” but deny active suicidality or a wish to die. Such statements can cause concern among geriatricians as well as family members and other clinicians working with older patients, who feel uncertain how to react or whether acute psychiatric care may be necessary. In our view, these attitudes are not fully accounted for by the well-established constructs of a neutral, approach or escape acceptance of death [13]. We sought to examine a FRD particularly within a sample of frail and/or ill “old-old” adults in a geriatric clinic, who were being acutely confronted with their own mortality and possible impending death. The goal of the present study was to examine to what extent a FRD is associated with or separate from other notions of death attitudes, including examining prevalence as well as correlates of a FRD within geriatric patients.

Our findings indicate that a majority of geriatric patients (70%) endorse an at least “somewhat” FRD with 29% agreeing “almost totally” that they hold a FRD. In contrast 30% rejected this concept completely. Moreover, endorsement of a FRD was significantly associated with several variables of interest, including greater death acceptance, as well as less death rejection and fear of death. A FRD was also associated with greater religiosity or spirituality, as well as a satisfied “hunger for life”. Importantly, although these factors were significantly correlated, associations remained weak (*r* <.30) for most variables. The largest correlation was with the “acceptance of one's own dying and death” subscale on the MODDI-F (*r* =.43), which also only accounted for 1*8*% of the variance in a FRD, suggesting no one variable alone accounts for a FRD. Importantly, a FRD suggests a positive connotation with death, which might distinguish it from neutral acceptance as measured in this study.

Direct comparisons of our results with previous work are limited as the concept of a FRD has not been utilized in previous studies. However, the relative frequent endorsement of a FRD is broadly in line with previous surveys indicating that most older adults accept death as a natural and unavoidable part of life [11, 12]. Considering that death avoidance is associated with poorer psychological well-being, whereas accepting attitudes have been shown to be associated with greater psychological well-being in older adults in previous work [43], it can be assumed that a FRD represents an adaptive, death-positive attitude. This is also broadly in line with concepts of emotion regulation in later life, specifically socioemotional selectivity theory (SST) [44] and Erikson’s psychosocial development model [12, 45, 46] indicating that many older adults have already attained a state of acceptance due to cognitive restructuring processes and changes in worldview, as well as adapting time perspectives, which consider the finitude of life [21, 45, 47, 48]. For the concept of a FRD, Meaning Management Theory may especially provide theoretical context regarding the possibility that older adults may hold positive associations with death as a satisfying end to a life well-lived. With regard to age-related physical changes, older adults adjust their expectations and values to align with their current capabilities. This adaptation helps sustain (emotional) well-being and ultimately fosters a greater sense of acceptance of aging [29, 49] and, consequently, death [29].

Individuals with a FRD appear to have recruited emotional coping strategies more effectively versus those without a FRD. Such differences are reflected in endorsement of the item “*My hunger for life is satisfied*.” Those with a FRD indicated a more “satisfied” hunger for life; however, interestingly, there were no significant group differences regarding the item “*I do not expect any more from life*.” This may suggest that a satisfied hunger for life represents a positive reflection on past experiences as well as a reduced drive, but not necessarily an indication that nothing more is expected from life. When older adults find meaning in their (past) life and evaluate their (previous) actions positively, this may also facilitate the acceptance of aging and, consequently, their own finitude [12]. The perception of having lived a fulfilled life promotes positive associations with death in later life, which in turn enhances well-being and supports the pursuit of new goals and meaning [12, 16].

Moreover, there is significant evidence that it is not chronological age itself, but rather the subjectively perceived proximity to death (future time limitation, FTL), which plays a crucial role in this inner, coping process in older age promoting positively toned readiness for death [28, 47]. Interestingly, whereas patients in our study were acutely ill, which may have induced an (implicit) FTL, responses to the question directly asking about life expectancy (“*I think I will live more than one year*.”) were not associated with a FRD and, on average, participants indicated believing they would live longer than one year. Although not significant, participants with a FRD, however, did endorse the item to a slightly less positive extent (indicating that they would not live more than one year). This may be clarified in future work.

Further, there were no differences based on FRD status in responses to self-developed items addressing time spent thinking about death and concerns about being remembered fondly after death. While these themes are emphasized in prevailing death acceptance frameworks (e.g., Wong’s approach acceptance, Erikson’s ego integrity, and Meaning Management Theory), their lack of association with FRD suggests that FRD may constitute a distinct construct. Moreover, the additional item about waiting for death without actively wishing for it also showed no significant correlation with FRD. This may, however, be attributable to insufficient operationalization and suboptimal item wording, as many participants indicated they were not “waiting” for death.

An important question in this exploratory work was whether a FRD is associated with physical ailments or frailty and whether it can be distinguished from mental illness and the active wish to die. Several studies have demonstrated that patients with depression and/or suicidality have a reduced neutral death acceptance and increased death anxiety [27, 50–52], whereas an increased acceptance of death as an escape from current suffering has also been associated with poorer physical and mental health [11] and would purportedly be associated with suicidality. Interestingly, in our study, a FRD was not correlated with suicidality or with our additional question addressing thoughts of killing oneself, and exhibited a negative, though non-significant, correlation with depression. Moreover, aspects commonly linked to suicidality or depression in older adults, such as overall disease burden [23, 31], cognitive functioning [31, 53], loneliness [31, 54] and frailty [55], were not associated with a FRD. Although it should be emphasized that suicidality was very low in our sample, these findings suggest that a FRD is not driven by depressive thoughts, suicidality, or the desire to escape current suffering due to physical illness. Rather, geriatric patients who report having come “to terms” with their past lives and maintain a friendly attitude toward death, without actively wishing for it, may lie outside the continuum from death wishes to passive or active suicidal ideation and have so far received little attention in geriatric medicine.

Finally, a FRD was associated with increased religiosity and/or spirituality, which is consistent with previous findings linking forms of death acceptance with both religious and non-religious forms of spirituality [10, 43, 50, 56]. There is evidence to suggest that religion and spirituality support finding meaning in late life, enhancing well-being, and fostering acceptance of death [10, 57, 58]. Considering that religiosity can serve as a defense mechanism against fear of death in older adults [27], religiosity and spirituality may serve as protective factors against negative attitudes toward death in old age and ultimately foster a more accepting and friendly outlook on death. Also reflected in our results is that this relationship appears to depend on the strength of intrinsic belief rather than the mere membership in a religious group [50]. It is important to note, however, that findings from different cultural contexts cannot be directly applied to our German sample. For instance, religiosity tends to play a more prominent role in the United States than in Germany [59]. Moreover, ethnicity can significantly shape older adults’ attitudes toward dying and death, including aspects such as religiosity [50]. To determine the relevance of religiosity in shaping a FRD within (German) older adults, mixed-method studies are necessary to capture a more nuanced understanding of this association.

### What is a friendly relationship to death?

Our findings suggest that a FRD is characterized by a preparedness for death in the absence of reduced expectations for what life may still hold in store. It is not linked (positively or negatively) to mental or physical illness, cognitive impairment or suicidal ideation and is apparently fostered by spirituality. These initial findings suggest it may represent an aspect of resilience or successful aging, reflecting an acceptance of one’s current phase of life, appreciation for and satisfaction with the life already lived and a positive relinquishment of the control of one’s fate. It lies between the acceptance of one’s mortality as a necessity (neutral acceptance) and the wish for death, to escape one’s current life (escape acceptance) or to reach a promised afterlife (approach acceptance) representing a not adequately explored, yet commonly endorsed, form of death acceptance.

### Clinical implications and future directions

Future research is needed to validate, define, and establish the construct of a FRD, and to examine whether assessment of this construct may provide relevant clinical information in the context of geriatric assessments. It may be associated with geriatric patients’ motivation for treatment compliance, readiness for life-sustaining medical interventions or wishes for assisted suicide. Decisions about life-sustaining treatment, for example, are complex and depend on many factors (e.g., type of intervention, duration of life extension, pain and mental health) [60]. Because preferences often fluctuate [61], whereas a prerequisite for the approval of assisted suicide (in Germany) is a sustained and “non-biased” wish to die [62], it would be important to explore whether a FRD contributes to these decision processes. Although there is evidence that death anxiety influences end-of-life choices [63], positive attitudes toward death have not, to our knowledge, been systematically examined in this context and would require careful investigation. This may also serve to enhance doctor-patient communication by improving provider knowledge of death acceptance. Finally, this line of research underscores the need for a broadened scope of and accessibility to palliative care, which provides compassionate end-of-life support in line with positive death attitudes and acceptance of mortality.

Beyond clinical decision-making, a FRD may also be relevant for broader models of aging. Resilience, meaning-making, and neutral death acceptance are crucial contributors to successful aging [9, 29, 54, 64]. Given evidence linking lower death anxiety to both successful aging [65] and greater readiness for death [9], a FRD could represent a positive dimension of resilience that supports well-being in later life. Particularly mixed-methods research utilizing qualitative interviews could help gain a more nuanced understanding of the phenomenology of FRD as well as the subjective experiences of older adults in various settings [66]. It also remains unclear how a FRD may evolve over the lifespan. It may be that, shortly before the end of life, when death becomes imminent, fear resurfaces. Wong states that we can never be entirely free from the fear of death and that fear and acceptance can indeed coexist [16].

### Limitations

Despite the many strengths of our study, including recruitment of a sample of frail, very old adults and use of standardized instruments, relevant limitations should be mentioned. Our study is based solely on cross-sectional data, which precludes causal conclusions, and it was not powered to detect differences with small effects. Given the exploratory nature of this work, results were not adjusted for multiple comparisons. Larger studies including longitudinal data collection may be warranted. It would be valuable to further investigate the concept of a FRD through additional qualitative research. Such studies could help to explore its associations with psychological constructs (e.g., defense mechanisms, intrapsychic conflicts, resistance) as well as with broader theoretical frameworks, such as Erikson’s theory of psychosocial development—particularly in relation to attitudes toward death and their development over time. Spirituality/religiosity was assessed with one item, and participants were not provided with an operational definition of these terms. Given the preliminary association with FRD, these constructs should be examined in more detail in future work.

Another potential limitation is that we assessed geriatric patients’ attitudes toward death in an early rehabilitation setting. These patients may have a more positive and recovery-oriented mindset compared to older adults in palliative care or hospice settings, whereas they may also have more immediate health concerns compared to those in senior residences or assisted living facilities. It is also unclear how these groups may differ from older adults with mental health problems (e.g., suicidality, depression) or healthy older adults. Future work should strive to examine FRD among older adults with diverse characteristics. It is also possible that patients with less positive attitudes toward death refused to participate in the study, leading to potential bias. Finally, the prevalence of suicidality and death wishes in our sample was low, so interpretations regarding associations between a FRD and suicidality warrant caution.

## Conclusion

Taken together, a positive relationship with the topic of death among geriatric patients appears to be prevalent and, if better understood and correctly assessed, a friendly relationship or preparedness for death could be seen not as a problematic phenomenon among older adults requiring psychiatric intervention but rather as a valuable aspect of successful aging and quality geriatric care.

## Supplementary Information

Supplementary Material 1.

## Data Availability

Data will be made available from the corresponding author (BS) upon reasonable request.
