# Supplementary material for: Exploring the concept of a “friendly relationship to death” in geriatric patients
Source: BMC Geriatr. 2025 Dec 19;26:61. doi: 10.1186/s12877-025-06670-6 (PMC12814579; doi:10.1186/s12877-025-06670-6)
Supplement: Supplementary file 1 — Supplementary Material 1. [file 12877_2025_6670_MOESM1_ESM.docx]

| **Supplementary Material Table 1.**  Spearman's correlation between a “Friendly Relationship to Death” and clinical characteristics | | | |
| --- | --- | --- | --- |
|  |  |  |  |
| Variable (*N* = 99) |  | *r* (*p*-value) | *% variance explained (r^2^)* |
| **Death attitudes (MODDI)** |  |  |  |
| Acceptance of one’s own dying and death (AODD) |  | **.43 (<.001)** | **18.5%** |
| Rejection of one’s own death (RODe) |  | **-.33 (<.001)** | **10.9%** |
| Fear of one’s own dying (FODy) |  | .02 (.872) |  |
| Fear of one’s own death (FODe) |  | **-.22 (.030)** | **4.8%** |
|  |  |  |  |
| **CFS** |  |  |  |
| Prior to admission |  | -.10 (.345) |  |
| Current |  | -.03 (.782) |  |
|  |  |  |  |
| **CIRS** |  | -.08 (.425) |  |
|  |  |  |  |
| **DIA-S** |  | -.19 (.055) |  |
|  |  |  |  |
| **BSS** |  | -.10 (.308) |  |
|  |  |  |  |
| **T-ILS** |  | .07 (.476) |  |
|  |  |  |  |
| **Additional questions** |  |  |  |
| “I often think about my death.” |  | .14 (.172) |  |
| “I think I will live longer than one year.”^a^ |  | -.06 (.557) |  |
| “I will be remembered fondly after my death.”^b^ |  | .09 (.383) |  |
| “I would describe myself as a religious or spiritual person.” |  | **.24 (.015)** | **5.0%** |
| “My hunger for life is satisfied.” |  | **.20 (.048)** | **4.0%** |
| “I do not expect any more from life.” |  | -.00 (.975) |  |
| “The thought of killing myself is not foreign to me.” |  | -.11 (.286) |  |
| “Here I wait for death without wanting or fearing it.” |  | **.30 (.003)** | **9.0%** |
| *Notes:*  ^a^ *N* = 96  ^b^ *N* = 98  CFS = Clinical Frailty Scale;  CIRS = Cumulative Illness Rating Scale;  DIA-S = Depression in Old Age Scale  BSS = Beck Scale for Suicide Ideation  T-ILS = UCLA Loneliness Scale (3-item version)  MODDI = Multidimensional Orientation Toward Dying and Death Inventory | | |  |
